# ROS signaling under metabolic stress: cross-talk between AMPK and AKT pathway

**DOI:** 10.1186/s12943-017-0648-1

**Published:** 2017-04-13

**Authors:** Yang Zhao, Xingbin Hu, Yajing Liu, Shumin Dong, Zhaowei Wen, Wanming He, Shuyi Zhang, Qiong Huang, Min Shi

**Affiliations:** Department of Oncology, Nanfang Hospital, Southern Medical University, Guangzhou, China

**Keywords:** Reactive oxygen species, Metabolic stress, AMPK, AKT, mTOR, FOXO

## Abstract

Cancer cells are frequently confronted with metabolic stress in tumor microenvironments due to their rapid growth and limited nutrient supply. Metabolic stress induces cell death through ROS-induced apoptosis. However, cancer cells can adapt to it by altering the metabolic pathways. AMPK and AKT are two primary effectors in response to metabolic stress: AMPK acts as an energy-sensing factor which rewires metabolism and maintains redox balance. AKT broadly promotes energy production in the nutrient abundance milieu, but the role of AKT under metabolic stress is in dispute. Recent studies show that AMPK and AKT display antagonistic roles under metabolic stress. Metabolic stress-induced ROS signaling lies in the hub between metabolic reprogramming and redox homeostasis. Here, we highlight the cross-talk between AMPK and AKT and their regulation on ROS production and elimination, which summarizes the mechanism of cancer cell adaptability under ROS stress and suggests potential options for cancer therapeutics.

## Background

Metabolic stress, prevalently existing in tumor microenvironments and characterized with nutrient, oxygen and growth factor deprivation, is the consequence of aberrant proliferation and relative inadequate angiogenesis and vascularization [1–4]. Glucose deprivation is one of the main patterns of metabolic stress due to the dramatic reliance on glucose for energy production in cancer cells [5, 6]. It is estimated that glucose concentration in tumors may be 3–10 folds lower than noncancerous tissues [7]. This nutrient deficiency directly reduces ATP production and leads to reactive oxygen species (ROS) overproduction [8].

The ROS accumulation activates multiple pathways and exerts discrepant impacts on cancer cell survival [9]. AMP-activated protein kinase (AMPK), the energy sensor in cells, is activated in response to this stress and promotes metabolic reprograming. AKT (also known as protein kinase B, PKB), a proto-oncogene activated in multiple cancers, acts as anti-apoptotic factor to a variety of stimuli such as radiation, hypoxia and chemotherapy [10]. However, growing number of studies indicate the activation of AKT does not inhibit cell death, but renders cells more sensitive to metabolic stress instead [11–14]. It is anticipated that anti-apoptotic ability of AKT is coupled with glucose metabolism. Glucose deprivation could induce ROS overload, causing AKT hyperactivation and accelerating cell death. This implies dual roles of AKT in tumor growth and stress resistance [11].

From the view of ROS production and elimination in combination with these two regulators under metabolic stress, we review the metabolic reprogramming and redox homeostasis of cancer cells, highlighting the cross-talk between AMPK and AKT and their influence on cancer progression and treatment.

### ROS-mediated cross-talk between energy and redox homeostasis

**Fig. 1:**
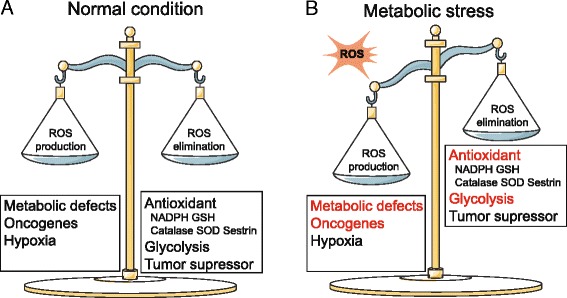
The cellular redox state is determined by ROS production and elimination: **a** Under normal condition, cancer cells maintain redox homeostasis by balancing ROS production and elimination. **b** Under metabolic stress, the redox homeostasis is damaged with enhanced ROS production and decreased ROS elimination

ROS are byproducts of biological reactions of energy generation, and are mainly produced in the mitochondria through the oxidative metabolism [15, 16]. It is estimated that ROS produced by mitochondria are about 1–2% of the total rate of oxygen consumption in normal cells [17]. Cancer cells prevalently exhibit much higher ROS levels than normal cells due to dysfunctional mitochondria, oncogene activation and antioxidant imbalance [8, 18]. ROS are a double-edged sword for oncogenesis. Moderate ROS inactivate the protein tyrosine phosphatases (PTP) such as phosphatase and tensin homolog (PTEN), facilitating phosphoinositide 3-kinase (PI3K) and tyrosine kinase receptor (TKR) signaling, which ultimately leads to tumor progression [19]. However, excess ROS damage cellular structures, such as the lipid membrane, protein and nucleic acid. Specifically for more proliferating cancer cells, excess ROS induce DNA mutations and compromise genome integrity, leading to cell senescence and death [18]. Cancer cells develop an antioxidant system comprised with ROS scavenging enzymes such as superoxide dismutases (SODs), catalase (CAT), glutathione peroxidases (GPX) as well as antioxidant agents like nicotinamide adenine dinucleotide phosphate (NADPH) and glutathione (GSH). The antioxidant capacities of cancer cells increase with the rising ROS levels, potentially as a survival adaptation [20–23]. The balance of ROS production and elimination maintains the cellular redox homeostasis, which is vital to cell survival (Fig. 1a).

Redox homeostasis is closely linked to glucose metabolism. Cancer cells make prevalent use of glycolysis to produce energy even in aerobic environments, a phenomenon known as the Warburg effect [24]. Although this mode of energy production is less efficient than mitochondrial respiration per unit of glucose, the rate of glycolysis is 10–100 times faster [25]. Glycolysis is an uncomplete energy release process and produces less ROS than mitochondria oxidation. The excess carbons from glycolytic intermediates are ingredients for biosynthesis of lipids, nucleic acids and proteins, which are necessary for increased de novo synthesis of cellular building blocks [26]. Increased glucose absorption is diverted directly or indirectly to pentose phosphate pathway (PPP) to produce NADPH [27], which further facilitates the conversion of oxidized glutathione (GSSG) to reduced GSH by acting as co-substrate of glutathione reductase (GR) [28]. The increased NADPH and GSH not only facilitate biosynthesis, but also create reductive milieu to resist oxidative stress [29]. In different phases of the cell cycle, the main energy-production methods may fluctuate between glycolysis and oxidative phosphorylation (OXPHOS). Glycolysis is a primitive method to produce energy in proliferating cells such as early embryonic cells, stem cells and cancer cells when compared to those rest cells. Enhanced glycolysis contributes to alleviating ROS stress and diminishing the chance of spontaneous mutation during DNA replication [30–32]. Therefore, glycolysis is a protective strategy for rapid ATP synthesis with less ROS stress in cancer cells [33].

### The conflicts of redox and metabolic homeostasis under metabolic stress

Under metabolic stress, the redox balance is damaged. Limited glucose sources impair glycolysis, and glycolysis-based NADPH production is depleted by reduced utilization of the PPP [34]. Additionally, glucose limitation leads to overburdening of mitochondria energy production. As a result, the metabolism rewires from glycolysis to OXPHOS and subsequent pro-oxidant production, primarily superoxide and hydrogen peroxide, leads to ROS overload [35, 36]. The disequilibrium of ROS production over ROS scavenging leads to ROS stress (Fig. 1b), which further activates apoptotic pathways. A study by NA Graham et al. based on phospho-tyrosine proteomics showed that metabolic stress provoked a supra-physiological level of TKR signaling, causing a positive feedback loop between ROS, PTPs and TKR signaling, and ultimately leading to cell death [37]. Meanwhile, by using ROS scavenger N-acetylcysteine (NAC), the kinase activation could be reversed under glucose deprivation [38, 39]. In addition, it is reported in M Gao’s study that Reverse Phase Protein Arrays (RPPA) analysis detected the signal change after glucose deprivation. A variety of kinases were activated, which was consistent with NA Graham’s study [37]. Interestingly, though the study of M Gao exhibited a dramatic difference in the spectrum of activated kinases between transient and prolonged glucose deprivation [40]. It was hypothesized that the kinase activation after glucose deprivation and its role in cell survival might be time-dependent and specific to cell lines. These studies underline the importance of the kinase activation loop in mediating ROS-induced cell death.

Although Otto Warburg, who proposed the theory of the Warburg effect, declares mitochondrial dysfunction leads to prevalent use of glycolysis in cancer cells, there are studies suggesting that many cancer cells prioritize OXPHOS to generate ATP [41]. It is generally agreed that enhanced glycolysis in cancer cells does not necessarily correspond to impaired OXPHOS [32]; particularly under metabolic stress, mitochondrial-based energy production is essential for viability. Under metabolic stress, other intermediates like glutamine [42, 43], lactate [44, 45], fatty acids and others [7, 46] are alternatively consumed to produce ATP. J Yun et al. reported that colorectal cancer cells demonstrated higher expression of KRAS and GLUT1 under glucose deprivation [47]. Additionally, glucose deprivation stimulated the tricarboxylic acid (TCA) cycle through mitochondrial glutamine metabolism, which was a source of ATP and generated a-ketoglutarate (α-KG) for biosynthesis [43, 48]. However, enhanced mitochondrial burden under metabolic stress increases the ROS production. The conflicts of producing energy versus maintaining ROS homeostasis dramatically impacts the fate of cancer cells.

### ROS regulation by AMPK and AKT under glucose deprivation

Glucose deprivation leads to ATP depletion and ROS accumulation, which in turn activates AMPK. AMPK is a heterotrimer complex, including a catalytic subunit (α) and two regulatory subunits (β and γ). It is phosphorylated on the Thr-172 in the presence of high AMP/ATP ratios due to ATP depletion, allowing it to act as an energy sensor for the cell. It is also regulated by its upstream LKB1, CaMMK or other factors like ADP or Ca^2+^ [49]. In addition, ROS directly activate AMPK through S-glutathionylation of cysteines on the AMPKα and β subunit [31].

**Fig. 2:**
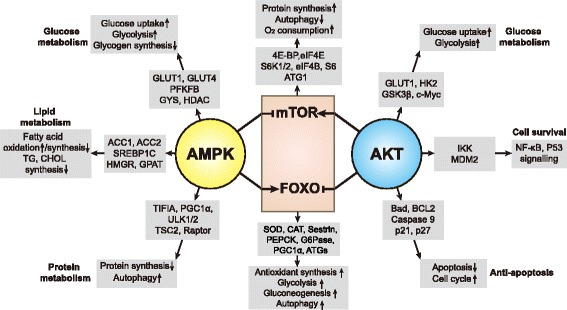
Cross effects of AMPK and AKT on the cellular metabolism and redox state: The targeted proteins regulated by AMPK and AKT and their regulatory effects are depicted, AMPK is a key player in response to metabolic stress by regulating the metabolism of glucose, lipid and protein. AMPK promotes glucose uptake and glycolysis, facilitating antioxidant production. AMPK also stimulates fatty acid oxidation and limits the fatty acid synthesis. mTOR and FOXO are two main downstream effectors of AMPK. AMPK inhibits mTOR activity, which induces protein synthesis inhibition and autophagy activation. AMPK also promotes FOXO activity to maintain the redox balance through enhanced antioxidant production and glucose metabolism. On the other side, AKT exerts antagonistic effect to regulate mTOR and FOXO activity. AKT stimulates mTOR signaling to promote glucose metabolism and protein synthesis, leading to increased ROS production. Meanwhile, it inhibits FOXO activity and renders cells susceptible to ROS toxicity

AMPK mediates metabolic reprogramming to survive glucose deprivation by promoting catabolism (glucose uptake, glycolysis, fatty acid oxidation, autophagy, etc.) and suppressing anabolism (protein, fatty acid, glycogen synthesis) [49–52] (Fig. 2). AMPK also regulates the redox state by alleviating the glucose deprivation-induced NADPH depletion via decreased fatty acid synthesis and increased fatty acid oxidation [46]. Further, B Chaube et al. found that AMPK could enhance mitochondrial biogenesis and OXPHOS by activating p38/PGC1α pathway [53]. This indicates AMPK is not only involved in glycolytic regulation, but also participates in ATP generation from OXPHOS.

The serine/threonine kinase AKT is widely acknowledged as a proto-oncogene. It is activated by extracellular signals (mostly growth factors through PI3K signaling) and is downregulated by PTEN. AKT mediates carcinogenesis and tumor progression mainly through promoting cell survival and inhibiting apoptosis [54] (Fig. 2). In addition, AKT is evolutionarily conserved and regulates glucose metabolism [14]. AKT promotes glycolysis through increasing GLUT1 trafficking to the cell surface, and through phosphofructokinase (PFK) and hexokinase (HK) activation [12]. Moreover, by stimulating oxidative metabolism, AKT promotes mitochondria oxygen consumption and contributes to ROS accumulation [55]. AKT is downstream to multiple growth factors such as EGF, IGF and HGF, etc. These growth factors are increased via autocrine or paracrine signals in nutrient-abundant conditions [56], indicating the role of AKT in proliferation is closely related to a well suitable growth milieu.

Recently, some studies indicate that the ability of AKT to inhibit cell death is dependent on glucose metabolism [13, 57]. JL Coloff et al. found that AKT suppressed Bim-induced cell death only when glucose was present [12]. Additionally, AKT activation rendered glioblastoma cells more sensitive to glucose withdrawal-induced cell death [13], and overexpression of PTEN dramatically reversed this process [37]. Further, V Nogueira et al. found that AKT activation rendered cells more susceptible to ROS-mediated premature senescence and cell death by increasing oxygen consumption and suppressing FOXO activity [14]. These studies imply that AKT acts as a pro-apoptotic factor under ROS stress, which is at odds with the established cognition of AKT as a tumor protective gene. Moreover, AKT is one of the factors involved in the aforementioned glucose deprivation-induced cell death via strengthening the kinase activation loop [37].

### The cross-talk between AMPK and AKT under metabolic stress

It is interesting that under glucose deprivation, AKT plays antagonistic roles from AMPK in ROS-mediated cell apoptosis. mTOR and FOXO are two main downstream effectors regulated by both AMPK and AKT, which exert antagonistic effects on ROS homeostasis. In addition, AMPK and AKT also regulate mutual phosphorylation directly or indirectly.

#### mTOR signaling

**Fig. 3:**
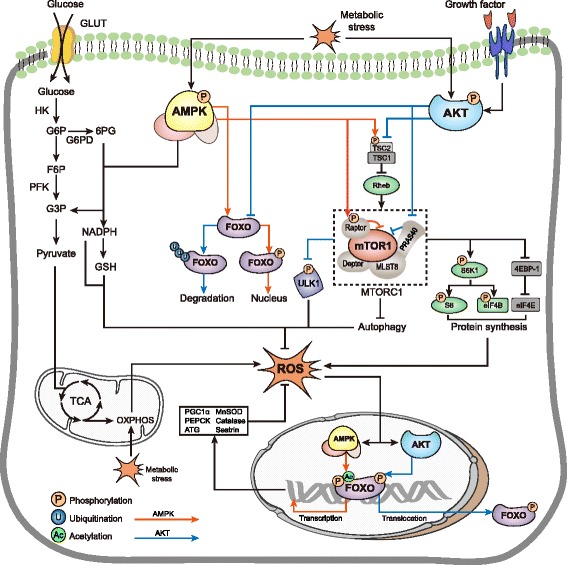
Signaling of AMPK and AKT on the ROS homeostasis via mTOR and FOXO regulation: Under metabolic stress, AMPK inhibits mTOR mainly via two ways:phosphorylates TSC2 at Ser-1387 which stimulates the TSC1-TSC2 complex to inhibit Rheb’s ability to activate mTOR; phosphorylates Raptor at Ser-792/Ser-722 to inhibit mTOR1. AKT activates mTOR reversely: AKT phosphorylates TSC2 at another site and activates mTOR via Rheb; AKT phosphorylates PRAS40 to inhibit its ability to suppress mTOR. Activated mTOR in turn promotes protein synthesis through S6K1 and 4E-BP1. AMPK phosphorylates FOXO, promoting the translocation to nucleus. AMPK also facilitates FOXO acetylation and enhances its transcriptional activity of antioxidant genes: SOD, Catalase, Sestrin. Additionally, AMPK promotes NADPH production via the PPP. On the other hand, AKT phosphorylates FOXO and leads to the translocation from the nucleus to the cytoplasm. By ubiquitination of FOXO, AKT leads to its degradation in cytoplasm

mTOR is a nutrient and growth factor sensing complex, which lies the intersection between glucose and amino acid metabolism and contributes to biosynthesis and autophagy [58]. Active mTOR1 phosphorylates ribosomal protein S6 kinase (S6K) and eukaryotic translation initiation factor 4E (eIF4E) binding proteins (4E-BPs), controlling the activity of eukaryotic initiation factors (eIFs) and eukariotic elongation factors (eEFs). This promotes the protein synthesis and ribosome biosynthesis [31, 58]. In addition, mTOR1 inactivates UNC­51­like kinase 1 (ULK1) at Ser-757 to inhibit autophagy. Interestingly, AMPK phosphorylates ULK1 at Ser-317 and Ser-777 to initiate Beclin1-mediated autophagy [59]. AMPK phosphorylates the regulatory-associated protein of mTOR (Raptor) at Ser-792/Ser-722 and tuberous sclerosis complex 2 (TSC2) at Ser-1387, indirectly leading to inhibition of mTOR1 (Fig. 3). mTOR1 activity is positively correlated with mitochondrial activity and promotes oxidative metabolism [60, 61]. Under glucose deprivation, AMPK inhibits mTOR1 activity, thereby decreasing protein synthesis and increasing autophagy. Decreased anabolism reduces ROS production, while enhanced autophagy and glycolysis increases the resilience of cells to ROS.

However, opposed to AMPK, AKT activates mTOR by inhibiting TSC2 and subsequently allowing Rheb-GAP to phosphorylate mTOR1. Additionally, AKT inactivates PRAS40, which alleviates the PRAS40-mediated inhibition of mTORC1 (Fig. 3). By activating mTORC1, AKT promotes oxygen consumption and increases ROS production under glucose deprivation [54, 62], rendering cancer cells closer to the death threshold of ROS.

#### FOXO signaling

FOXO acts in response to starvation and oxidative stress. FOXO normally exists in cytoplasm in an inactive form and translocate to nucleus to initiate transcriptional activity once activated [63]. FOXO activation increases resistance to oxidative stress by targeting the expression of SOD, catalase and sestrin, which are the antioxidant enzymes that maintain redox homeostasis [64]. In addition, FOXO participates in glucose metabolism by regulating phosphoenolpyruvate carboxykinase (PEPCK), Glucose-6-phosphatase (G6Pase) and peroxisome proliferator-activated receptor γ coactivator 1α (PGC1α). PEPCK is the key enzyme in gluconeogenesis which is enhanced in energy deprivation and promotes glucose and glutamine metabolism [65, 66]; PGC1α is a major transcription coactivator closely related to mitochondria biogenesis and OXPHOS [67], PGC1α positive cells exhibit increased ROS detoxification capacities in some cancers such as melanoma [68]. In addition, FOXO induces expression of autophagy-related genes (ATG6, ATG7, ATG12, etc.) to elevate autophagic flux and increases the production of mainly fatty acid and amino acids consumed by mitochondria OXPHOS [64, 69].

AMPK can directly regulate FOXO. AMPK enhances FOXO3-mediated transcriptional activity by recruiting CREB-binding protein (CBP) and p300 [63, 70, 71]. It also regulates S-phase kinase-associated protein 2/coactivator-associated arginine methyltransferase 1 (SKP2/CARM1) signaling by FOXO3 phosphorylation to induce autophagy under glucose starvation [72]. In EL Greer’s study, AMPK-mediated FOXO3 phosphorylation did not affect the nuclear localization of FOXO3 [73], indicating AMPK influences FOXO3 activity only when it is in the nucleus. Conversely, there are also conflicting studies that suggest that AMPK may in fact facilitate FOXO3 nuclear localization [64, 74]. The exact effect of FOXO phosphorylation remains unclear. In addition, AMPK may increase nuclear FOXO acetylation and mediate localization to nuclear promyelocytic leukemia (PML) bodies, which act as transcriptional co-activators [75]. AMPK indirectly promotes FOXO acetylation by retaining class II histone deacetylase (HDAC) in the cytosol [63, 76]. Taken together, FOXO post-translational modifications greatly influence its function and AMPK is crucial in this process (Fig. 3).

In contrast to AMPK, FOXO is negatively regulated by AKT signaling. Glucose deprivation-derived ROS production induces the nucleus translocation of FOXO and thereby promotes transcriptional activity of antioxidant-related genes [63, 77]. However, AKT inhibits this process by phosphorylating the FOXO at three conserved residues and inversely translocates FOXO from nucleus to cytoplasm [78]. Besides, AKT also promotes the ubiquitination of FOXO and leads to its degradation [79] (Fig. 3).

#### Mutual phosphorylation regulation of AMPK and AKT

AKT blunts AMPK activation. In a rat model of ischemia perfusion, AKT phosphorylates AMPKα1/α2 at Ser-485/491 (equivalent to Ser-487/491 in human), while the phosphorylation of Thr-172 is reduced [80]. In another rat model of hypoxia, AKT activation also prevents the phosphorylation of AMPKα at Thr-172 [81]. This phenomenon is consistently found in human normal tissues as well as in tumor such as breast and liver cancer cell [82–85]. Ser-487 of AMPKα is located in the serine/threonine-rich loop (ST loop, residues 472–525 in human) within the C-terminal domain of AMPKα1, which interacts with residues within the kinase domain. Ser-487 phosphorylation hinders the ability of upstream kinase LKB1 or CaMMK to access Thr-172 [83]. Moreover, in specific glioblastoma and breast cancer cells characterized by hyperactivity of AKT due to loss of PTEN, AMPK is resistant to activation by AMPK activator A769662, though this effect is reversed by addition of MK2206 as an AKT inhibitor [83, 86].

**Fig. 4:**
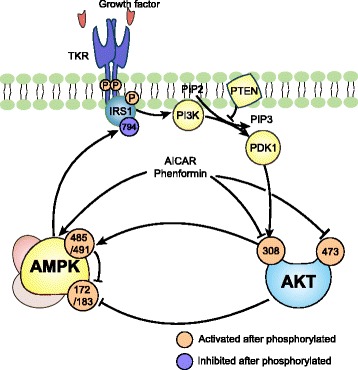
Interaction between AMPK and AKT on the phosphorylation: Growth factor activates TKR and promotes the activation of AKT via IRS1/PI3K/PDK1 signaling. Activated AKT phosphorylates AMPKα on Ser-485/491, preventing the active site Thr-172 to get access to LKB1 or CaMMK. AMPK phosphorylates IRS1 at Ser-794 and inhibits AKT signaling. AMPK activated by AICAR or phenformin dephosphorylates Ser-473 and Thr-308 of AKT, inhibiting AKT activity

AMPK reversely inhibits AKT phosphorylation. AMPK activated by AICAR or phenformin dephosphorylates Ser-473 and Thr-308 of AKT, thereby inhibiting its activity. This blockade of AKT by AMPK agonists consequently lowers the inhibition of AKT’s downstream effector, glycogen synthase kinase-3α/β (GSK3α/β) [87]. In addition, AMPK affects AKT signaling by regulating insulin receptor substrate 1 (IRS1), which is phosphorylated by the insulin receptor and mediates PI3K activation. AMPK phosphorylates IRS1 at Ser-794 (in human, equivalent to Ser-789 in rats) and inhibits AKT signaling [88–90]. However, there are studies indicating AMPK sensitizes AKT phosphorylation through IRS signaling [91]. It is reported that activated AMPK by AICAR stimulates AKT through the same phosphorylation site of Ser-789 [86, 92, 93]. It seems that IRS1 phosphorylation at Ser-794 in humans (Ser-789 in rats) has dual roles in AMPK-mediated AKT signaling, and its function needs to be further determined [93–95]. Taken together, AMPK and AKT have mutual complicated antagonism, which may partially explain their roles in metabolism and redox maintenance (Fig. 4).

In general, mTOR inhibition and FOXO activation is essential in AMPK-mediated metabolic reprogramming and ROS scavenging to maintain the redox hemostasis under metabolic stress. On the other hand, AKT activation renders cells more sensitive to ROS-mediated cell death by impairing redox homeostasis through opposite regulation of mTOR and FOXO from AMPK. Additionally, AMPK and AKT have mutual regulation on phosphorylation. These antagonistic regulations are significant in maintaining redox homeostasis under glucose deprivation.

### The regulation of AMPK and AKT in response to glucose supply

When glucose is abundant, AMPK activity remains limited and AKT is relatively activated, promoting cancer cell growth, division and metastasis. In addition, growth factor autocrine or paracrine signaling under suitable milieu forms positive feedback loops to activate AKT. Activated AKT also promotes the production of ROS, which stimulates oncogenic pathway, leading to uncontrolled proliferation. However, higher ROS levels render cells closer to the threshold of ROS lethality, which is regarded as the Achilles’ heel of AKT [14].

**Fig. 5:**
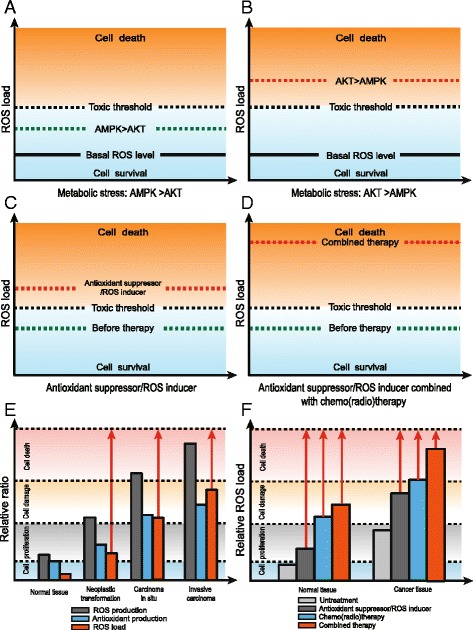
AMPK and AKT mediate ROS regulation in tumor progression and treatment: **a**-**b** Under metabolic stress, AMPK and AKT manipulate ROS regulation and influence cell survival. The basal ROS indicate the ROS level under normal condition. **c**-**d** ROS load is increased by inducing ROS production or suppressing antioxidants. The effects are magnified when combined with chemo/radiotherapy. **e** ROS and antioxidant capacities are increased with tumor progression. **f** Compared to normal tissues, cancer cells are more susceptible to ROS targeting therapy, chemo/radiotherapy or their combined application

Under glucose deficiency when AMPK predominates, it resists against glucose deprivation-derived ROS accumulation by increasing glycolysis and the PPP. Meanwhile, autophagy and OXPHOS are enhanced to balance the input and output of energy, relieving the ROS load. This is achieved by the downstream effectors of AMPK, of which FOXO activation and mTOC1 inhibition play key roles (Fig. 5a).

Under glucose deprivation when AKT predominates, the anti-apoptotic role of AKT is reversed since glucose is lacking. AKT inhibits FOXO translocation to the nucleus and decreases antioxidant production. Unlike AMPK, which is ubiquitously activated under glucose deprivation, AKT seems to be different among cancer cells. AKT is activated in glioblastoma and Hela cells, for example [13, 14], but inhibited in ovarian cancer and leukemic T cells [12, 96]. In addition, PTEN significantly influences AKT activity under glucose deprivation. When PTEN is present in lung cancer cells, AKT phosphorylation is increased after glucose deprivation. When PTEN is mutated or knocked down, AKT phosphorylation is inhibited instead [97]. This suggests that AKT activation is context-dependent among cell lines and decided by multiple factors. There are reports suggesting that AKT activation can protect cells under glucose deprivation. It is found that site-specific phosphorylation of AKT at site Thr-308 decreased cell death [40]. This shows that under glucose deprivation, AKT function is sophisticated and specific to different cancer cells and backgrounds (Fig. 5b).

### The role of ROS regulation through AMPK and AKT in tumor progression

#### Anoikis resistance

Glucose deficiency is one of the main factors leading to ROS stress in tumors, either in the stage of tumor initiation or progression [18]. At the initial stage of metastasis, cancer cells must leave its original niche to enter the vascular or lymphatic circulation. This entails detaching from the extracellular matrix (ECM) and becoming anchorage independent, which leads to ATP deficiency owing to reduction of glucose transport [22]. In normal human cells, this transition leads to a strong induction of ROS, which leads to anoikis. Tumor cells, on the other hand, may become anoikis resistant and successfully leave their primary niche to become circulation tumor cells (CTC) and facilitate metastatic colonization [98].

Under ECM-detached condition, AMPK promotes survival by autophagy induction and global inhibition of protein synthesis, mitigating the ATP reduction [99, 100]. AMPK also enhances the PPP and increases the NADPH production. Addition of antioxidants like Trolox and NAC can rescue ATP deficiency independent of glucose uptake, further demonstrating the role of AMPK in anoikis resistance [22]. In addition, AKT is activated upon ECM detachment by TKR activation, and it inhibits cell death mainly by promoting glucose uptake and upregulating anti-apoptotic pathways such as BCL2 signaling. AMPK and AKT are both activated in the resistance of anoikis, but AMPK activation seems to be dominant. It is reported that mTOR, the downstream of both AMPK and AKT, was inhibited in aniokis resistant cells [99, 101, 102].

#### Drug resistance

Many chemotherapy agents and radiotherapy kill cancer cells through ROS-mediated cell death, and ROS resistance is one way for cancer cells to develop drug resistance. Tumor stem cells, which are considered the seed of relapse, have superior resistance to anti-tumor agent [103]. Due to enhanced antioxidant systems, these cells tend to have a lower ROS load than to their non-tumorigenic progeny [18, 104, 105]. Increased autophagy is important for chemotherapy resistance. AMPK, as the inhibitor of mTOR, has been reported to induce autophagy-mediated drug resistance [106–108]. Additionally, replenishing NADPH through AMPK-mediated glucose uptake and the PPP also contributes to drug resistance.

#### Targeting ROS as anti-cancer therapy

Even though cancer cells exhibit an enhanced antioxidant system, they still maintain higher ROS levels than normal cells [20, 109]. ROS load increases with the tumor progression [23]. Compared to normal cells, cancer cells are closer to the threshold of ROS toxicity. Interrupting redox homeostasis may be a potential target to inhibit tumor metastasis and mitigate the drug resistance. This could be achieved by inhibition of ROS detoxification or stimulation of ROS production [18] (Fig. 5c-f).

Metabolic inhibition is one way to cause ROS accumulation. 2-deoxyglucose (2-DG), the glucose analogue, competes with the glucose transporter and inhibits HK2 activity and classically used to mimic metabolic stress. 2-DG not only activates AMPK, but also induces AKT phosphorylation [110, 111]. It is designed to have anti-tumor activity both in vivo and in vitro, but the clinical trials fail to reach better patient outcomes after a single use of 2-DG [112]. However, 2-DG combined with chemotherapy or radiotherapy has had better results, as it renders cancer cells more sensitive to apoptosis by further elevation of ROS [113, 114]. The inability of AKT to inhibit ROS-mediated cell apoptosis also provides a strategy to treat cancer. In tumor with hyperactivated AKT, further ROS production sensitizes cells to ROS induced apoptosis. This has been achieved by combined use of ROS inducer phenylethyl isothiocyanate (PEITC) and mTOR inhibitor rapamycin, which completely eradicate tumor growth in cells with hyperactivated AKT both in vitro and in vivo [14]. Since mTOR elicits a negative feedback loop to suppress AKT, rapamycin could lead to further AKT activation through mTOR1 inhibition [115]. In addition, RV Pusapati et al. found that 2-DG-induced glycolytic inhibition could be enhanced by active mTOR signaling via increased glutamine uptake and pentose phosphate flux [111], which suggested metabolic inhibition alone was not sufficient for cancer treatment. Promisingly, the combination of glycolytic inhibition and ROS inducer may exert a more synergistic effect for cancer therapy.

Depletion of antioxidant is another way to induce ROS toxicity. GSH is the most abundant antioxidant in cells and its recyclability is dependent on NADPH production. Targeting GSH dramatically breaks down the redox balance, as demonstrated by the dramatic inhibition of the thioredoxin (TXN) pathway by the combination use of buthioine sulfoximine (BSO) and auranofin (AUR) [28]. PEITC, an inhibitor of glutathione peroxidase (GPX), also depletes GSH and shows anti-tumor effects in multiple cancers. Further, sulphasalazine (SSA) decreases GSH levels by inhibiting the cysteine transport, reducing the growth and viability in cancer cells [116, 117]. A newly synthetized enzyme cysteinase could effectively lead to ROS elevation and cell death by depleting extracellular L-cysteine, which is essential for cellular GSH synthesis [118]. However, there are controversies about antioxidant depletion to treat cancer. Previous studies stress that antioxidant can decrease carcinogenesis and tumor development [119], but recent studies show that antioxidant can relieve the anoikis-mediated oxidative stress and promote cell survival and metastasis [120–122]. Some antidiabetic drugs like saxagliptin, sitagliptin and antineuropathic α-lipoic acid (ALA) also have antioxidant properties, and are reported to promote metastasis by increasing antioxidant capacity [123].

## Conclusions

During cancer progression, cancer cells are frequently confronted with metabolic stress, which is accompanied with redox disequilibrium. The rewiring of glucose metabolism and antioxidant maintenance intersect at the response to the stress. In this process, AMPK and AKT play significant roles. AMPK mediates metabolic change to resist the ROS accumulation, while AKT renders cells more susceptible to ROS mediated cell death. This review summarizes the cross-talk between these two kinases, mainly through antagonistic regulation on their downstream effectors, mTOR and FOXO. We suggest that under metabolic stress, the role of AMPK and AKT might be varied and context-specific to influence the cell fate.

The tight relationship between the AMPK and AKT acting on ROS homeostasis is closely related to the tumor progression and treatment. The resilience of ROS toxicity is one of the main mechanisms of tumor metastasis and drug resistance. Studying the dynamic change of ROS in tumor progression deepens the understanding of aniokis and metastatic colonization. Further, modulation of oxidative stress by targeting ROS detoxification or stimulation provides new strategies to treat cancers. Compared to traditional chemotherapy or radiotherapy, combined utilization of ROS inducer (or antioxidant inhibitors) with traditional therapies may exert synergistic effect through enhanced ROS-mediated cell death. Meanwhile, this strategy may cause less harm to normal cells, which have more potential capacities to resist ROS than cancer cells. Therein, targeting ROS may be a promising way for anticancer therapy, and their regulatory mechanisms need more detailed research.

## Abbreviations

2-DG: 2-deoxyglucose
4E-BP: Eukaryotic translation initiation factor 4E-binding protein
6PG: 6-phosphogluconate
ACC: Acetyl-CoA carboxylase
AICAR: 5-aminoimidazole-4-carboxamide1-β-D-ribofuranoside
AKT: Protein kinase B
ALA: α-lipoic acid
AMPK: AMP-activated protein kinase
ATG: Autophagy-related gene
AUR: Auranofin
BAD: Bcl2-associated agonist of cell death
BSO: Buthioine sulfoximine
CARM1: Coactivator-associated arginine methyltransferase 1
CAT: Catalase
CBP: CREB-binding protein
CTC: Circulation tumor cell
Deptor: DEP domain-containing mTOR-interacting protein
ECM: Extracellular matrix
eIF4B/E: Eukaryotic translation initiation factor 4B/E
F6P: Fructose-6-phosphate
FOXO: Forkhead box protein O
G3P: Glycerol-3-phosphate
G6P: Glucose-6-phosphate
G6Pase: Glucose-6-phosphatase
G6PD: Glucose-6-phosphate dehydrogenase
GLUT: Glucose transporter
GPAT: Glycerol phosphate acyltransferase
GPX: Glutathione peroxidase
GR: Glutathione reductase
GSH: Glutathione
GSK: Glycogen synthase kinase
GSSG: Oxidized glutathione
GYS: Glycogen synthase
HDAC: Histone deacetylase
HK: Hexokinase
HMGR: 3-hydroxy-3-methylglutaryl-CoA reductase
IKK: NF-kappa-B essential modulator
IRS1: Insulin receptor substrate 1
LKB1: Liver kinase B1
MDM2: E3 ubiquitin-protein ligase Mdm2
MLST8: Target of rapamycin complex subunit LST8
mTOR: Mammalian target of rapamycin
NAC: N-acetylcysteine
NADPH: Nicotinamide adenine dinucleotide phosphate
OXPHOS: Oxidative phosphorylation
PDK1: 3-phosphoinositide-dependent protein kinase 1
PEITC: Phenylethyl isothiocyanate
PEPCK: Phosphoenolpyruvate carboxykinase
PFK: ATP-dependent 6-phosphofructokinase
PFKFB: 6-phosphofructo-2-kinase/fructose-2,6-biphosphatase
PGC1α: Peroxisome proliferator-activated receptor γ coactivator 1α
PI3K: Phosphoinositide 3 kinase
PK: Pyruvate kinase
PML: Promyelocytic leukemia
PPP: Pentose phosphate pathway
PRAS40: Proline-rich AKT1 substrate of 40 kDa
PTEN: Phosphatase and tensin homolog
PTP: Protein tyrosine phosphatases
Raptor: Regulatory associated protein of mTOR
ROS: Reactive oxygen species
RRPA: Reverse phase protein array
S6K: Ribosomal protein S6 kinase
SKP2: S-phase kinase-associated protein 2
SOD: Superoxide dismutase
SREBP1C: Sterol regulatory element-binding protein 1C
SSA: Sulfasalazine
ST loop: Serine/threonine-rich loop
TCA: Tricarboxylic acid
TIFIA: Transcription initiation factor IA
TKR: Tyrosine kinase receptor.
TSC2: Tuberous sclerosis 2
TXN: Thioredoxin
ULK: UNC-51-like kinase
α-KG: α-ketoglutarate
